# In vitro comparison of the leakage of carbon dioxide and iodine contrast media in a bleeding model

**DOI:** 10.1186/s42155-024-00457-3

**Published:** 2024-05-10

**Authors:** Ryoichi Kitamura, Kazuhiro Yoshida, Takaaki Maruhashi, Satoshi Tamura, Yutaro Kurihara, Koyo Suzuki, Yasushi Asari

**Affiliations:** 1 Department of Emergency and Critical Care Medicine, Kitasato University School of Medicine, 1-15-1, Kitasato, Minami-ku, Sagamihara, Kanagawa 252-0375 Japan; 2 Department of Medical Engineering and Technology, Kitasato University School of Allied Health Sciences, Kanagawa, Japan; 3 Department of Emergency Medicine, Kitasato Medical Center Hospital, Saitama, Japan

**Keywords:** Carbon dioxide, CO_2_, CO_2_ angiography

## Abstract

**Background:**

We aimed to compare the hydrodynamic values of carbon dioxide (CO_2_) and iodine contrast media for bleeding detection using an in vitro model.

**Materials and methods:**

We created a bleeding model with large and small wounds in simulated blood vessels. We connected a syringe to the bleeding model and the blood pressure transducer, filling the circuit with CO_2_ and iodine contrast media. The syringe’s piston was pressed, and the flow rate and intravascular pressure of the CO_2_ and iodine contrast media leaking from the bleeding model were measured. We compared each leaked contrast medium’s volume, sphere-equivalent diameter, and sphere-equivalent area. These values were analyzed to compare the visibility of the leakage objectively.

**Results:**

At a constant flow rate, the intravascular pressure required for the model to leak was lower for the CO_2_ than that for the iodine contrast medium. The CO_2_ contrast medium leakage volume, equivalent circle diameter, and equivalent circle area were greater than those of the iodine one. These values indicate higher CO_2_ visibility during fluoroscopy.

**Conclusions:**

In the bleeding model, a CO_2_ contrast medium may be more prone to leakage than the iodine one in large and small wounds. Regarding visibility, a CO_2_ contrast medium may be more likely to detect leakage than an iodine one.

## Background

Carbon dioxide (CO_2_) angiography is widely used as an alternative method for patients in whom iodine contrast media cannot be used, such as those with iodine allergies or renal dysfunction [1]. CO_2_ angiography is reportedly effective in identifying sources of bleeding that cannot be detected with iodine contrast medium during the endovascular treatment of bleeding, such as gastrointestinal and obstetric bleeding [2, 3]. When performing angiography with an iodine contrast medium, as in the case of gastrointestinal bleeding, identifying the bleeding point is challenging. In persistent massive active bleeding cases, detecting the source of bleeding is relatively easier, even with iodine-contrast angiography. However, in clinical practice, bleeding can be intermittent, and even if a catheter is inserted into a bleeding vessel, extravasation cannot be detected if the bleeding rate is below the detectable threshold. Low viscosity is a well-known characteristic of CO_2_. Owing to its low viscosity, CO_2_ is sensitive enough to detect extravasation, and CO_2_ angiography is effective in identifying the source of bleeding, even when it cannot be determined by angiography with an iodine contrast medium [2]. However, fundamental studies on CO_2_ angiography are rare, and few have examined iodine contrast medium and CO_2_ in detail [4]. In this study, a bleeding model was created using a vascular model to compare the hydrodynamics of CO_2_ and iodine contrast media to understand the effect of CO_2_ in increasing the sensitivity of bleeding detection.

## Methods

### Bleeding modeling using vascular models and experimental setup

**Fig. 1:**
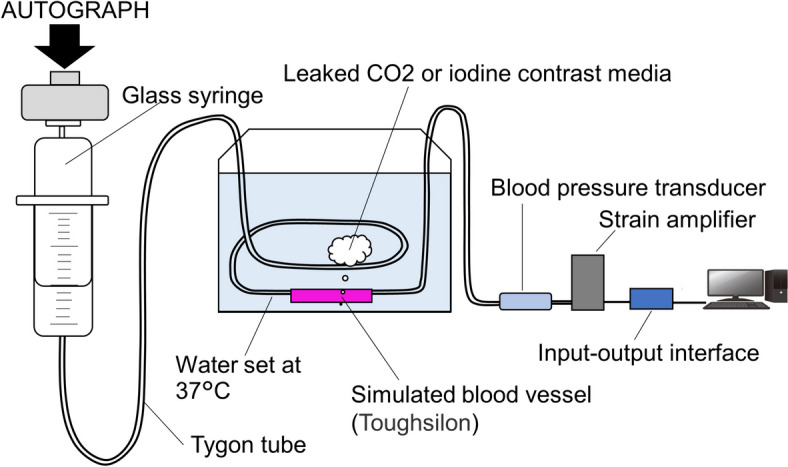
Schematic diagram of the experimental apparatus. The simulated blood vessels with large and small wounds were connected to glass syringes using TYGON tubes. A bleeding model and blood pressure transducer were used to monitor the blood pressure. The output voltage from the strain amplifier was transferred to the load tester via an input/output interface and recorded using the load tester control application. All circuits were filled with iodine contrast medium or CO_2_. A glass syringe was placed on the autograph to measure the pressure when CO_2_ or iodine contrast medium leaked from the simulated blood vessel

Figure 1 shows a schematic of the experimental apparatus. Bleeding models were created by perforating the center of a simulated blood vessel (Toughsilon TSG-A10; TANAC, Gifu, Japan) with an inner and outer diameter of 4 and 6 mm, respectively and a length of 50 mm using a syringe needle. The effect of wound size was examined by defining it as significant when perforated with an 18-gauge needle and small when perforated with a 27-gauge needle. A glass syringe (Tsubasa Industry, Tokyo, Japan) and the bleeding models were connected using Tygon tubes (Yamato Scientific) with an inner diameter of 4 mm. A blood pressure transducer (DX-100; Nihon Kohden, Tokyo, Japan) was connected to the bleeding model to monitor the intravascular pressure. The blood pressure transducer was connected to a strain amplifier (CDV-700A; KYOWA ELECTRONIC INSTRUMENTS, Tokyo, Japan), and the output voltage from the strain amplifier was input into a load-testing machine (AUTOGRAPH AGS-X; SHIMADZU, Kyoto, Japan) via an input-output interface (SENSOR I/O EXTENSION BOX; SHIMADZU) and recorded using a load-testing machine control application (TRAPEZIUMX; SHIMADZU). Glass syringes, Tygon tubes, bleeding vessel models, and blood pressure transducers were filled with an iodine contrast medium (Omnipaque; GE Healthcare, Japan) or CO_2_. Tygon tubes and bleeding models were fixed at the bottom of a 37 °C thermostatic bath to account for changes in viscosity with temperature. The height of the water surface was fixed at 8 cm, assuming an intra-abdominal pressure. Iodine or CO_2_ contrast media were injected into the bleeding models using a load tester to maintain the piston of the glass syringe at a constant speed and flow rate. An experimental system was created in which the perforation of the bleeding model was the only exit from the contrast-filled circuit.

### Experimental preparation and protocols

**Fig. 2:**
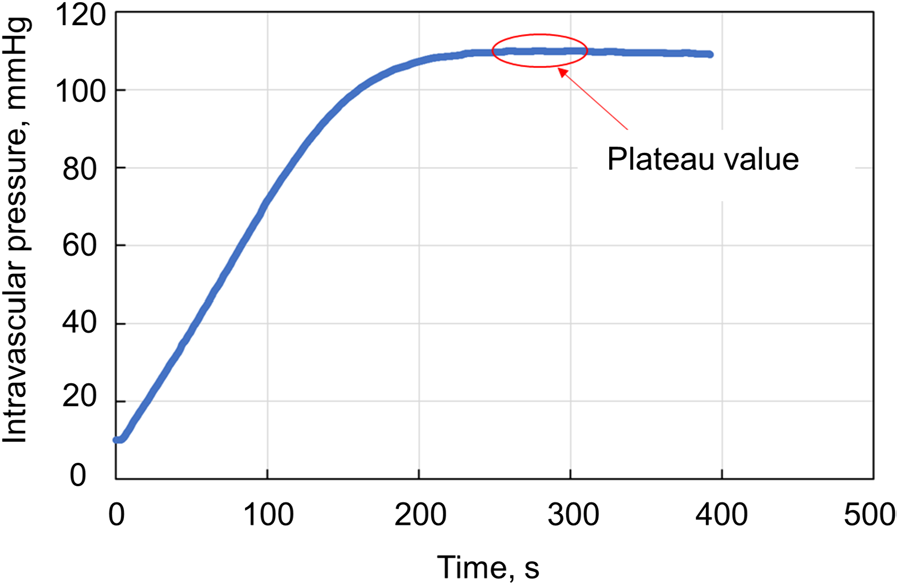
Pressure variation over time when pumping at a constant speed and flow rate. It was observed that the pressure gradually increased after the start of the test and became constant after a short time. When the expansion of the tubing and the flow of the contrast medium were stabilized, the pressure in the circuit was constant, and the pumping and bleeding flow rates were equal. The stabilization pressure was measured as the pressure at which a constant flow rate leaked

During a preliminary experiment using the aforementioned experimental setup, changes in the intravascular pressure over time during pumping at a constant speed and flow rate were recorded (Fig. 2). After the start of the injection, the intravascular pressure gradually increased and then remained steady, a phenomenon confirmed by reproducibility. The intravascular pressure at the stabilization time was measured when the leakage flow rate was constant. CO_2_ is considered a compressible fluid; therefore, it expands after leaking out of the bleeding model. When detecting bleeding points using a radiographic fluoroscopy system, obtaining the leakage flow rate extended outside the bleeding model is necessary to determine the amount of iodine contrast medium or CO_2_ that leaks outside the vessel. Therefore, the leakage flow rate of CO_2_ expanding out of the vessel was corrected using Boyle’s law. The bleeding model was assumed to be an abdominal visceral artery, and the flow rate was corrected for an atmospheric pressure of 760 mmHg and an intra-abdominal pressure of 6 mmHg. The equation relating the leakage flow, intravascular pressure in the contrast medium, and wound size was determined using the least-squares method. To compare the visibility of the radiographic fluoroscopy system, the leaked iodine or CO_2_ contrast media were calculated in a two-dimensional sphere, and its diameters were compared. If the fasting blood flow rate of the superior mesenteric artery is 200 mL/min and one shot of iodine contrast medium or CO is 20 mL, the time required for the iodine contrast medium or CO_2_ to pass through the bleeding point is 0.1 min. Therefore, the leakage flow rate (mL/min) at a given blood pressure can be obtained from the relational formula for leakage flow and intravascular pressure of the iodine contrast medium or CO_2_ and multiplied by 0.1 min to get the volume of iodine contrast medium or CO_2_ leaking out of the vessel in one iodine contrast medium or CO_2_ shot at a given blood pressure. We calculated the equivalent circular diameter and the equivalent circular area at a blood pressure of 100 mmHg.

### Evaluation items

We obtained equations relating leakage flow to intravascular pressure, Pearson’s correlation coefficient, and the coefficient of determination for iodine contrast medium or CO_2_ in small or large wounds. JMP Pro 17 software (SAS Institute, Tokyo, Japan) was used for the statistical analysis, with a significance level of 5%. The volume, sphere-equivalent diameter, and sphere-equivalent area of the leaked iodine or CO_2_ contrast media in small and large wounds were compared using the iodine contrast medium as a control.

## Results

**Fig. 3:**
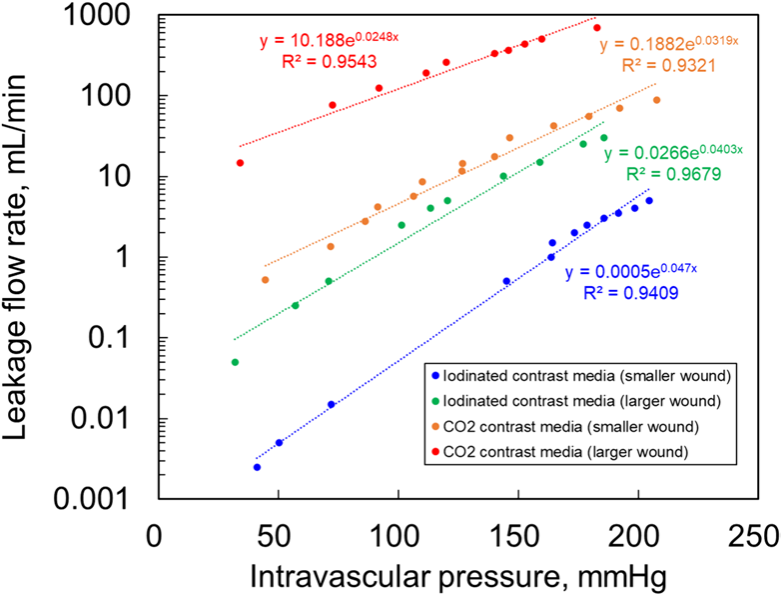
Relationship between iodine contrast medium and CO_2_ leakage flow and intravascular pressure. The flow rate leaking out of the vessel under constant intravascular pressure was higher with CO_2_ than with iodine contrast medium and CO_2_ and even more significant with more extensive wounds. Leakage flow was higher when CO_2_ was used in the simulated vessel with a small wound than when an iodine contrast was used in the simulated vessel with a large wound

**Fig. 4:**
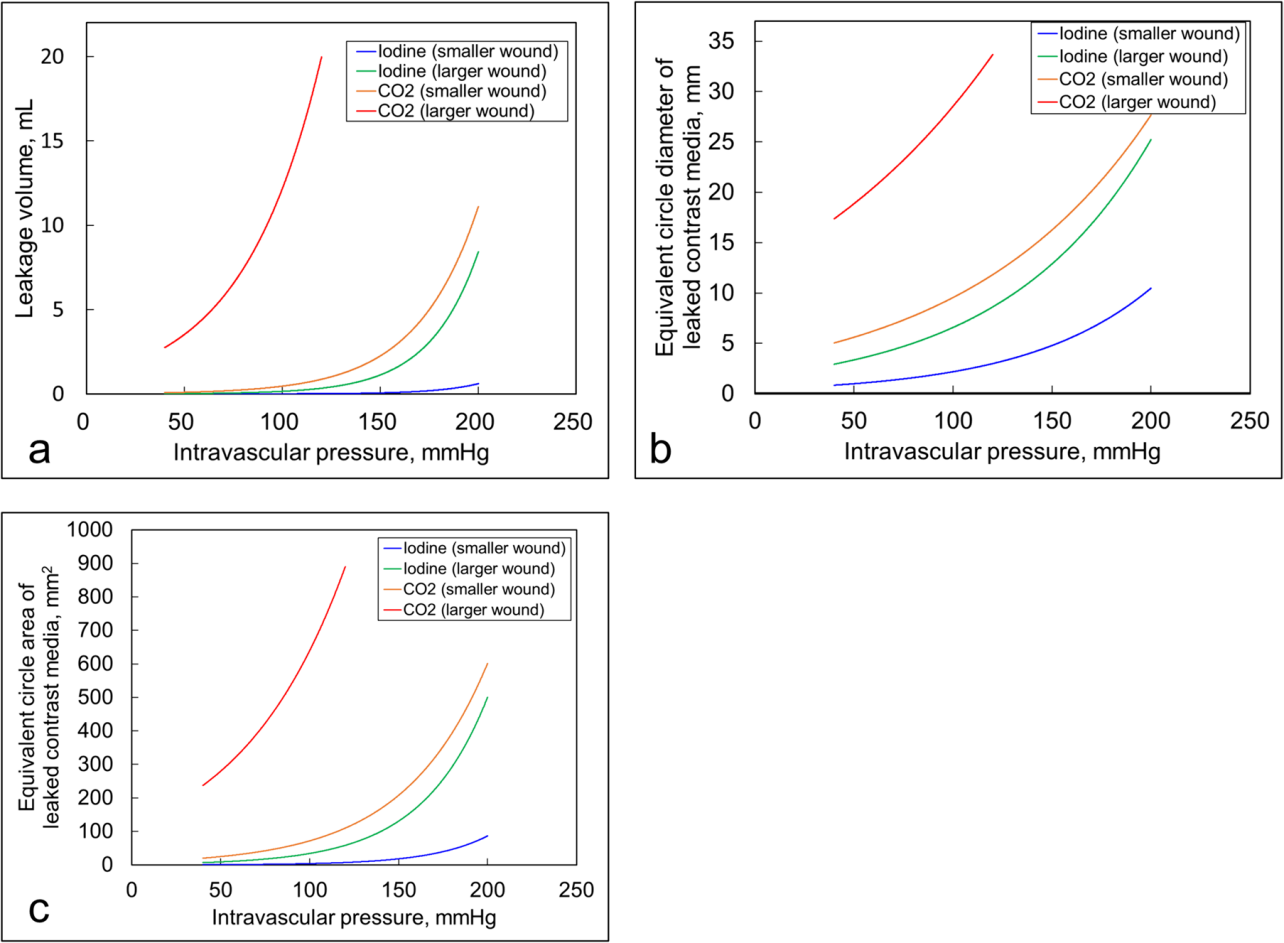
**a** Relationship between intravascular pressure and leakage volume. **b** Relationship between intravascular pressure and equivalent circular area. **c** Relationship between intravascular pressure and an equivalent circular area of leaked iodine contrast medium or CO_2_. The leakage volume, equivalent circle diameter, and equivalent circle area were also greater for iodine and CO_2_, with larger values for more extensive wounds

**Fig. 5:**
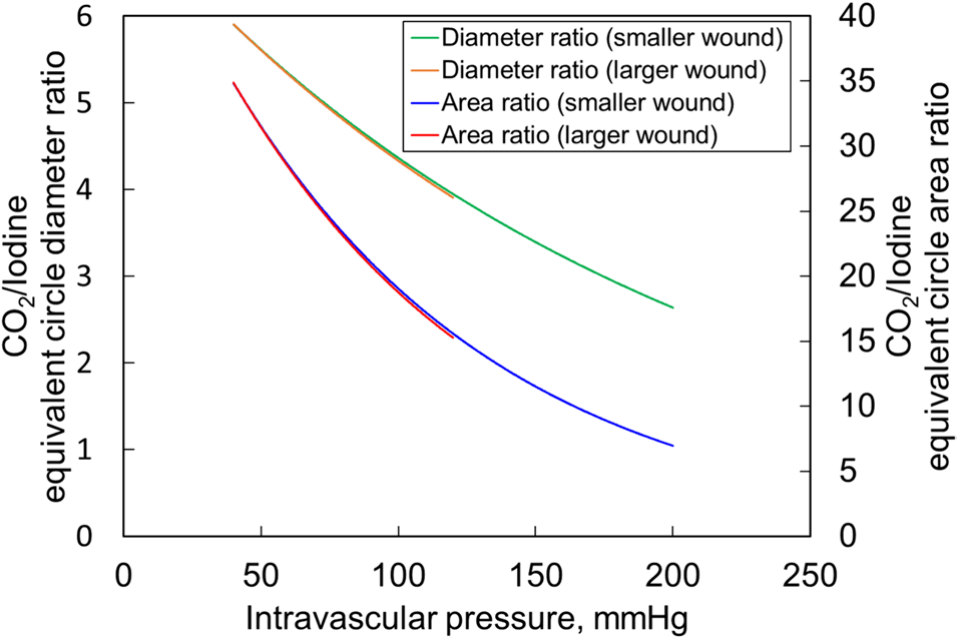
The relationship between intravascular pressure and the CO_2_/iodine contrast medium ratio of the equivalent circle diameter as well as that of intravascular pressure and the CO_2_/iodine contrast medium ratio of the equivalent circle area. The CO_2_/iodine contrast medium ratio for the equivalent circle diameter and the CO_2_/iodine contrast medium ratio for the equivalent circle area were higher at lower intravascular pressures and similar for the large and small wounds

The relationship among the iodine contrast medium, CO_2_ leakage flow rate, and intravascular pressure is shown in Fig. 3. Significant differences in the correlation coefficients were observed for all the conditions (*p* < 0.0001), yielding high coefficients of determination (*R*^2^ > 0.95). The flow rate of the CO_2_ leaking out of the vessel at constant intravascular pressure was higher than that of the iodine contrast medium, and the flow rate increased as the wound size increased. At a constant intravascular pressure, the leakage flow rate was higher when CO_2_ was used in the bleeding model with a small wound than when iodine contrast medium was used in the bleeding model with a large wound. When the intravascular pressure was 100 mmHg, the leakage flow rate of the iodine contrast medium was 0.05 mL/min for small wounds and 4.52 mL/min for CO_2_, which was approximately 90 times higher. The volume, equivalent circle diameter, and area of the iodine or CO_2_ contrast media leaking out of the bleeding model by one shot of iodine or CO_2_ contrast media that were calculated from the equation relating the leakage flow rate and intravascular pressure obtained from Fig. 3, are shown in Fig. 4a/b/c. The relationship between intravascular pressure and the CO_2_/iodine contrast medium ratio of the equivalent circle diameter and that of intravascular pressure and the CO_2_/iodine contrast medium ratio of the equivalent circle area is shown in Fig. 5.

At a blood pressure of 100 mmHg, the leakage volume for iodine contrast medium was 0.005 mL and 0.15 mL for small and large wounds, respectively, whereas for CO2 contrast, it was 0.457 mL and 12.1 mL for small and large wounds, respectively. The equivalent circle diameter for iodine contrast medium was 2.19 mm and 6.59 mm for small and large wounds, respectively, compared to 9.56 mm and 28.5 mm for small and large wounds with CO2 contrast, respectively. The equivalent circle area was 3.77 mm^2 and 34.08 mm^2 for small and large wounds, respectively with iodine contrast medium, whereas it was 71.7 mm^2 and 639.5 mm^2 for small and large wounds, respectively with CO2 contrast. The leakage volume, equivalent circle diameter, and equivalent circle area were also more critical for iodine contrast medium and CO_2_, with larger values for more significant wounds. Assuming a blood pressure of 100 mmHg, the diameter of CO_2_ leaking from a small wound was approximately 4.4 times larger than that leaking from the iodine contrast medium, and the projected area of the leaked CO_2_ was visible as a circle approximately 19.1 times larger than that of the iodine contrast medium. The CO_2_/iodine contrast ratio for the equivalent circle diameter and the CO_2_/iodine contrast medium ratio for the equivalent circle area was higher at lower intravascular pressures, and the large and small wounds had similar values.

## Discussion

This study showed CO is more prone to leakage than iodine contrast medium. The flow rate and volume of contrast leakage in the bleeding models were greater with CO_2_ than with iodine. The fluoroscopic images used in actual clinical practice are two-dimensional, and the area of CO_2_ or iodine contrast medium leakage, which reflects the ease of viewing, was found to be approximately 20 times larger for CO_2_ than for iodine contrast medium.

In this study, the leakage flow rate was more significant for CO_2_ than that for the iodine contrast medium, which the low viscosity of CO_2_ may explain. Poiseuille’s law states that the flow rate in a conduit between two points is inversely proportional to viscosity; thus, lower viscosity indicates higher flow rate from the bleeding point. The blood viscosity varies according to its hematocrit, usually 3–4 cPa.S (at 37 ℃ and 1 atm). The iodine contrast medium commonly used in angiography is estimated to be 9.1–10.6 cPa.S (at 37 ℃ and 1 atm), whereas the viscosity of CO_2_ is 0.0013 cPa.S (at 37 ℃ and 1 atm), < 1/1000th of that of blood. Therefore, CO_2_ may have a higher leakage flow rate than an iodine contrast medium or blood.

The leakage volume was also more significant for CO_2_ than the iodine contrast medium. Liquids change their overall volume only slightly as they move from high-to low-pressure environments, whereas gases increase in volume as they move to low-pressure environments. Iodine contrast medium, a liquid, does not change much in volume when it leaks, whereas CO_2_, a gas, expands in low-pressure environments according to Boyle’s law and is therefore likely to develop in volume when it leaks. The threshold for bleeding detection in conventional angiography using iodine contrast medium has been reported to be 0.5–1.0 mL/min [5, 6]. The results of the present study also show that the area of contrast leakage, which is associated with visibility, was more significant for the CO_2_ contrast medium than for the iodine contrast medium. Assuming a blood pressure of 50–150 mmHg, the leakage flow rate of the iodine contrast medium is generally < 2.0 mL/min, whereas the flow rate of CO_2_ is higher than that of the iodine contrast medium. This may allow the detection of undetectable extravascular leakage with iodine contrast medium in clinical practice.

A previous case series of acute lower gastrointestinal bleeding showed that CO_2_ angiography effectively identifies the source of bleeding when iodine contrast is ineffective [2]. In contrast, in a case series on CO_2_ angiography in acute gastrointestinal bleeding, the authors concluded that CO_2_ was inferior to iodine contrast in detecting gastrointestinal bleeding [7]. This is because they performed CO_2_ angiography in the celiac, superior mesenteric, and inferior mesenteric arteries. In contrast, Sawada et al. performed CO_2_ angiography in the right colic and sigmoid arteries with smaller vessel diameters [2]. The lumen of the tube and bleeding models used in our study was 4 mm, similar to the diameters of the vessels used in the study by Sawada et al. [2]. This result indicates the usefulness of CO_2_, as reported by Sawada et al. [2].

These results also indicate the possibility of detecting bleeding leaks in clinical practice using CO_2_ in endovascular treatment for bleeding in cases where an iodine contrast medium cannot be used to identify the bleeding site.

The present study had several limitations. First, it is entirely “in vitro”, and significant anatomical differences are frequently faced in real clinical cases. For example, suppose the vessels were filled with contrast medium only, mixing and lysis with blood were not considered. In that case, the vessels were not circulating vessels or beating flow, and the meandering of the vessels was not considered. Second, in actual clinical practice, the mechanism of hemostasis involves vascular spasm and thrombus formation and not simply a hole in the wound. Therefore, verifying the results of this study through ex vivo experiments in animals using similar experimental systems is necessary.

## Conclusions

In vitro experiments using a bleeding model showed CO_2_ was more prone to leakage than iodine contrast medium in flow and volume. Visibility also suggests that CO_2_ is more likely to detect leakage than the iodine contrast medium.

## Data Availability

The data associated with this research are available from the corresponding author upon reasonable request.

## Abbreviation

CO_2_: Carbon dioxide
